# UBE2D3 is a positive prognostic factor and is negatively correlated with hTERT expression in esophageal cancer

**DOI:** 10.3892/ol.2015.2926

**Published:** 2015-02-03

**Authors:** GE GE GUAN, WEN BO WANG, BING XIN LEI, QIAO LI WANG, LIN WU, ZHEN MING FU, FU XIANG ZHOU, YUN FENG ZHOU

**Affiliations:** 1 Hubei Key Laboratory of Tumor Biological Behaviors, Hubei Cancer Clinical Study Center, Wuhan University, Wuhan, Hubei 430071, P.R. China; 2 Department of Radiation and Medical Oncology, Zhongnan Hospital, Wuhan University, Wuhan, Hubei 430071, P.R. China

**Keywords:** ubiquitin-conjugating enzyme E2D 3, human telomerase reverse transcriptase, esophageal cancer, prognosis

## Abstract

Human telomerase reverse transcriptase (hTERT) is a critical factor in unlimited cell proliferation and immortalization, with numerous studies demonstrating that high expression of hTERT is a poor prognostic factor in various types of cancer. Ubiquitin-conjugating enzyme E2D 3 (UBE2D3) is a member of the E2 family, and participates in the ubiquitin proteasome pathway to regulate basic cellular activities, such as cell cycle control, the DNA damage response, apoptosis, and tumorigenesis. Our previous study initially determined that downregulation of UBE2D3 expression increases hTERT expression and cell proliferation, however, the association between the expression of these two proteins and their functions in cancer tissues remains unknown. Therefore, the protein expression levels of hTERT and UBE2D3 were evaluated in 150 esophageal cancer and 30 adjacent healthy tissue samples by performing immunohistochemical analysis. Concurrently, the clinicopathological data of the enrolled patients were obtained to allow correlation analysis. It was identified that the expression of hTERT in the esophageal cancer tissues was significantly higher compared with that of the adjacent tissues (P=0.015), however, the expression of UBE2D3 was significantly lower in esophageal cancer tissues than the adjacent tissues (P=0.001). Additionally, the study demonstrated that hTERT was significantly upregulated in poorly-differentiated, advanced tumor-node-metastasis (TNM) stage cancer tissues (P<0.05 for all), however, UBE2D3 expression was downregulated in poorly-differentiated, lymph node invaded cancer tissues and recurrent cases. It was also identified that traditional factors, including tumor location, T stage, lymph node status, TNM stage, and molecular factors of hTERT and UBE2D3, were significantly associated with overall survival time (P<0.05 for all). Furthermore, UBE2D3, lymph node status and tumor location were independent prognostic factors for esophageal cancer in multivariate analysis. Most notably, hTERT and UBE2D3 expression were negatively correlated with each other. In conclusion, the findings of the present study indicated that hTERT and UBE2D3 proteins appear to be involved in the development of esophageal cancer, that UBE2D3 may a positive prognostic factor for esophageal cancer, and that UBE2D3 and hTERT expression levels are inversely correlated.

## Introduction

Esophageal cancer is the eighth most common type of cancer and ranks as the sixth leading cause of cancer-related mortality worldwide (1,2). In particular, China contains areas of the highest incidences of esophageal cancer in the world, with esophageal cancer classified as the sixth most frequently diagnosed type of cancer (3) and the fourth most common cause of cancer mortality in China (4). In the previous two decades, comprehensive therapeutic regimes aimed at improving survival have been widely applied, however, the overall five-year relative survival rate is just 16.9% (5) due to the aggressive nature of this type of malignancy. Currently, the traditional tumor-node-metastasis (TNM) classification systems of the International Union Against Cancer (UICC) and American Joint Committee on Cancer are the most important tools used for predicting the prognosis of esophageal cancer patients and the only method used on a routine basis (6). However, esophageal cancer is highly heterogeneous, and tumors with the same TNM stage demonstrate differences in clinical course and treatment response. Thus, the identification of factors to predict malignant potential and prognosis is of great importance.

Telomeres are unique structures composed of double-stranded tandem repeats of TTAGGG at the end of a chromosome (7). Telomerase, a ribonucleoprotein enzyme, consists of a catalytic protein unit, human telomerase reverse transcriptase (hTERT), and human telomerase RNA. Together, the two units synthesize telomeric sequences that allow tumor cells to escape from cellular senescence when reactivated or are upregulated by tumor cells to allow indefinite proliferation (8). The expression of hTERT closely correlates with telomerase activity and serves as an indicator of telomerase activation (9,10). Although previous studies have demonstrated that high hTERT expression is a predictive and prognostic biomarker of a poor outcome in a range of malignancies, including Ewing’s sarcoma, and colorectal, gastric and breast carcinoma (11–14), studies regarding the role of hTERT expression in esophageal cancer are sparse and thus warrant further investigation.

The ubiquitin proteasome pathway (UPP) and the lysosome degradation system are two distinctive proteolytic mechanisms required for intracellular protein degradation (15). The UPP participates in protein quality control via the degradation of misfolded proteins (16) in order to regulate basic cellular activities, such as cell cycle control, the DNA damage response, apoptosis and tumorigenesis (17). The functioning of the UPP is achieved by the coordinated action of a cascade of three enzyme species: Ubiquitin activating enzymes (E1), ubiquitin-conjugating enzymes (E2) and ubiquitin ligases (E3) (16,18). Ubiquitin-conjugating enzyme E2D 3 (UBE2D3), also known as UBCH5C, is a member of the E2 family. In our previous study, a yeast two-hybrid screen was performed to identify that a mutual association exists between hTERT and the hTERT-interacting protein UBE2D3; downregulation of UBE2D3 resulted in the accumulation of hTERT, indicating that UBE2D3 potentially has a role in the hTERT signaling pathway (19). Thus, the aim of the present study was to elucidate the clinical significance of hTERT and UBE2D3 expression in esophageal cancer.

## Patients and methods

### Patients and follow-up

The esophageal cancer tissue specimens were obtained from patients who had undergone curative surgery (esophagectomy with lymph node dissection) at the Zhongnan Hospital of Wuhan University (Wuhan, Hubei, China) between January 2006 and December 2012. No patients had previously received palliative resection, pre-operative chemotherapy or radiotherapy, and no patients exhibited synchronous or metachronous cancer in other organs or succumbed in the month following surgery. All post-operative specimens were diagnosed with esophageal cancer by two pathologists and the TNM stage was determined according to the seventh edition of the UICC TNM system (20).

According to the aforementioned criteria, 150 patients were included in the present study. Follow-up data were available for all patients on a one to three month basis, the most recent follow-up date was June 10, 2014, and the median follow-up time was 37 months (range, 14–90 months). In addition, a total of 91 (60.7%) patients succumbed during the follow-up period. Any recurrence in the anastomotic mediastinum, esophagus or regional lymph nodes was defined as local-regional recurrence, and recurrence identified via blood flow was defined as distant metastasis, such as liver, lung and bone metastasis. Overall survival (OS) time was defined as the period between the date of the surgical procedure and the date of mortality. Furthermore, the present study was conducted in accordance with the Declaration of Helsinki of the World Medical Association and the protocols were approved by the Ethics Committee of Zhongnan Hospital of Wuhan University, with consent obtained from all patients.

### Immunohistochemical analysis and evaluation

Immunohistochemical staining was performed using the streptavidin-biotin method to detect hTERT and UBE2D3 protein expression levels. Firstly, the 4 μm-wide sections of esophageal cancer and adjacent tissues were incubated at room temperature for 60 min, followed by exposure to dimethylbenzene for 10 min. The sections were then deparaffinized in 100, 95 and 75% ethyl alcohol for 5 min, respectively. Next, the tissue samples were microwaved in citrate buffer (0.1 mol/l, pH 6.0) for 10 min at 1,000 W. Endogenous peroxidase was blocked by incubating the sections in 3% hydrogen peroxide for 5 min at room temperature. Subsequent to washing with distilled water and incubating with goat serum (Fuzhou Maixin Biotechnology Development Co., Ltd., Fuzhou, China) for 1 h, the sections were incubated with primary monoclonal hTERT (cat no., ab32020; dilution, 1:100; Abcam, Cambridge, MA, USA) or polyclonal UBE2D3 (cat no., 11677–1-AP; dilution, 1:50; Proteintech Group, Inc., Chicago, IL, USA) antibodies at room temperature for 2 h. Biotinylated secondary goat anti-rabbit antibodies and peroxidase-conjugated streptavidin obtained from the DAKO Universal LSAB™ kit (Dako, Glostrup, Denmark) were applied for 20 min each. Finally, the sections were incubated in 3′3′-diaminobenzidine for 5 min, followed by hematoxylin counterstaining. Two negative controls were performed by replacing the primary antibody with non-immune serum and omitting the application of the secondary antibody.

The intensity of the immunostaining was evaluated using light microscopy. Two independent investigators, who were blinded to the clinicopathological data, evaluated the levels of protein expression in each section, and the staining intensity was scored as follows: No staining, 0; weak staining, 1; moderate staining, 2; and strong staining, 3. Additionally, the extent of staining was scored according to the percentage of positively-stained cells in the entire carcinoma-involved area, as follows: Absent, 0; sporadic, 1–10%; local, 11–25%; occasional, 26–50%; majority, 51–75%; and large majority, 76–100%. For hTERT expression, an intensity score of ≥2 and ≥11% of cells with positive hTERT staining was considered to indicate high expression, and an intensity score of <2 or <11% of cells with hTERT positive staining was considered to indicate low expression (21). By contrast, UBE2D3 protein expression levels were classified as high when UBE2D3 staining was present in >50% of cells (22). Expression in the cytoplasm and nucleus was regarded as an indication of positive expression for the two proteins (22,23). Using this method, the expression of hTERT and UBE2D3 was detected in 150 esophageal cancer and 30 adjacent tissue samples.

### Statistical analysis

Statistical analyses were performed using SPSS software (version 17.0; SPSS, Inc., Chicago, IL, USA). The Pearson χ^2^ test or Fisher’s exact test were used to compare qualitative variables. Kaplan-Meier analysis was performed for univariate analysis and significance among patient subgroups was calculated using the log-rank test. Furthermore, the Cox proportional hazard model was used to conduct multivariate analysis and Spearman correlation coefficients were applied for analyzing the association between the expression of the two proteins. Two-sided P<0.05 was considered to indicate a statistically significant difference.

## Results

### Major clinicopathological features and immunohistochemical findings

Included in the present study were 145 samples of squamous cell cancer and five other pathological types. The age range of the patients was 39–85 years (mean ± standard deviation, 60.0±9.42 years). Additional clinical data for the current patients are presented in Table I.

**Figure 1:**
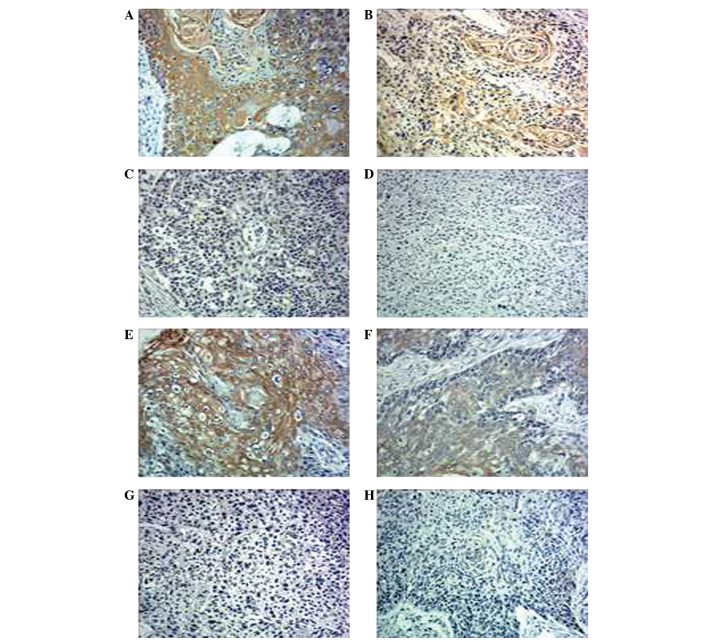
Immunohistochemical staining of human telomerase reverse transcriptase (hTERT) and ubiquitin-conjugating enzyme E2D 3 (UBE2D3) protein in esophageal cancer tissues. Representative examples of (A–B) high and (C–D) low hTERT expression, and (E–F) high and (G–H) low UBE2D3 expression (magnification, ×100).

Immunohistochemical analysis of all esophageal cancer and healthy adjacent tissues was performed. According to the aforementioned classification system, 11 healthy adjacent tissue samples with high hTERT expression and 21 with high UBE2D3 expression were identified, compared with 91 esophageal cancer tissue samples with high hTERT expression and 55 with high UBE2D3 expression. Thus, the expression levels of these two proteins demonstrated a significant difference between the cancerous and adjacent tissues (P=0.015 and P=0.001, respectively). Fig. 1A–D shows representative examples of hTERT expression in esophageal cancer samples and Fig. 1E–H shows representative examples of UBE2D3 expression. According to the aforementioned staining intensity and extent scores, Fig. 1A, B, E and F demonstrated high expression levels, however, Fig. 1C, D, G and H demonstrated low UBE2D3 expression levels.

### Association between expression levels of hTERT and UBE2D3, and various clinicopathological features

As summarized in Table II, hTERT expression was significantly higher in larger-sized, poorly-differentiated, and advanced T, N and TNM stage tumors (P=0.039, P=0.032, P=0.030, P=0.018 and P=0.012, respectively). Furthermore, UBE2D3 expression levels were significantly lower in poorly-differentiated, advanced N stage and recurrent cases of esophageal cancer (P=0.007, P=0.016 and P=0.008, respectively).

### Survival analysis

**Figure 2:**
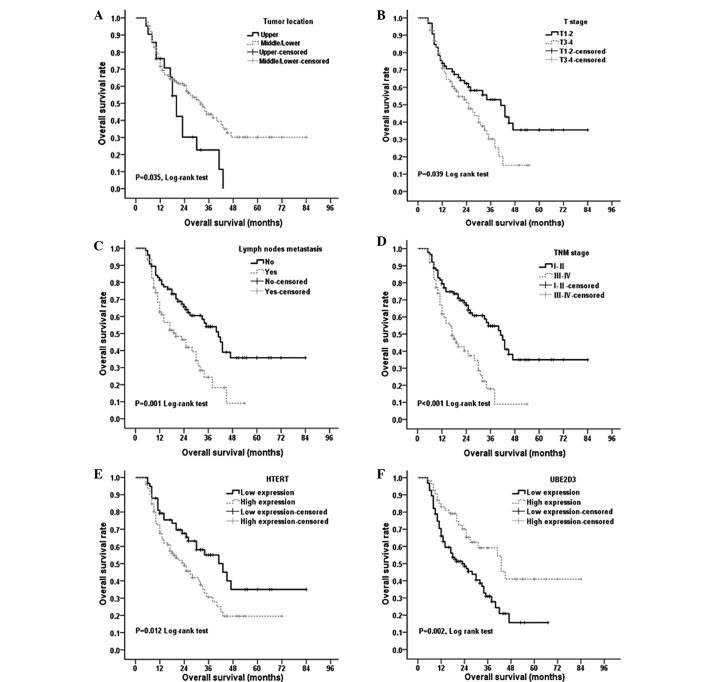
Cumulative overall survival (OS) of esophageal cancer patients. (A) A higher tumor location was associated with poorer OS. (B–D) Advanced T stage, lymph node status and TNM stage were associated with poor OS. (E) Patients in the high hTERT expression group were at a higher risk of mortality. (F) Patients in the UBE2D3 low expression group were at a higher risk of mortality. TNM, tumor-node-metastasis; hTERT, human telomerase reverse transcriptase; UBE2D3, ubiquitin-conjugating enzyme E2D 3.

In the 150 cases, the median OS time was 20.0 months (range, 5–84 months), and the one-, three- and five-year survival rates were 72.3, 40.7 and 25.0%, respectively. As indicated in Fig. 2, a number of traditional factors were associated with the OS time of the esophageal cancer patients, including tumor location, fibrous membrane invasion, lymph node status and TNM stage (P<0.05 for all; Fig. 2A–D). In addition, low expression levels of hTERT and high expression levels of UBE2D3 were associated with improved OS time (P=0.012 and P=0.002, respectively; Fig. 2E and F).

### Multivariate analysis

Univariate analysis (Table III) indicated that tumor location, T, N and TNM stage, and hTERT and UBE2D3 expression may predict esophageal cancer prognosis, therefore, these factors were integrated into multivariate analysis using Cox proportional hazards analysis. It was subsequently identified that tumor location, lymph node status and UBE2D3 expression level were independent prognostic factors in esophageal cancer (Table IV).

### Correlation between hTERT and UBE2D3 expression levels

The Spearman correlation coefficient for hTERT and UBE2D3 expression was r=-0.18 (P=0.027), indicating that hTERT and UBE2D3 expression are negatively correlated with each other.

## Discussion

The present study investigated 150 esophageal cancer and 30 adjacent tissues to evaluate the prognostic value of a number of conventional clinicopathological and cellular molecular factors. In accordance with previously conducted studies (24–26), univariate analysis demonstrated that traditional clinical parameters, such as T, N and overall TNM stage, were important factors for predicting the survival rate, whereas tumor histology and adjuvant therapy were not (Table II). However, whether tumor location affects the prognosis of esophageal cancer is a controversial topic. Yang *et al* (27) demonstrated that the tumor location did not impact the survival rate of esophageal cancer; however, in the seventh edition of the UICC TNM system (20), tumor location (upper and middle thoracic versus lower thoracic) was important for grouping T2-3N0M0 squamous cell cancers. The present univariate and multivariate analysis indicated that tumor location (upper versus middle and lower thoracic) was associated with survival rate and may be an independent prognostic factor in esophageal cancer. In addition, the present study identified that lymph node involvement may be an independent prognostic factor for esophageal cancer, which was consistent with the results of previous studies (28,29).

hTERT confers limitless replicative potential to cancer cells (30), and previous studies have established immortalized human esophageal epithelial cell models by the introduction of hTERT (31). Furthermore, hTERT is able to promote the development of invasive esophageal squamous cell cancer by interacting with epidermal growth factor receptor and p53 (32). Telomerase activity has been extensively studied in various types of malignant tumor for clinical, diagnostic and/or prognostic purposes (12,13,33), and it has been proposed for use as a marker of poor prognosis in such tumors. The present study determined that hTERT was more frequently elevated in the esophageal cancer tissues compared with the adjacent healthy tissues. In the cancer tissues, the expression of hTERT was also elevated in tumors with large size, poor differentiation, deep tumor invasion, lymph node metastasis and advanced TNM stage. Furthermore, strong expression of hTERT was correlated with OS time, indicating that hTERT participates in the progress of esophageal cancer and may be a poor prognostic biomarker of esophageal cancer tumors. However, in multivariate analysis, hTERT expression was not an independent prognostic factor, therefore, a combination test of telomerase activity with other prognostic factors may be necessary.

UBE2D3 is a member of E2 family and is a crucial component of the ubiquitination cascade, acting as a key mediator of the interaction between E1 and E3 (34,35). The whole ubiquitination process is responsible for 80% of proteasomal cellular protein degradation. Upregulation of UBE2D3 in acute promyelocytic leukemia cells leads to the ubiquitination of cyclin D1 and its degradation in the proteasome (36). However, in the absence of UBE2D3, cyclin D1 is not degraded and tumor cells continue to cycle (37). Mittal *et al* (38) reported that knocking down UBE2D3 in human breast cancer cells resulted in elevated cyclin D1 levels, and that a low level of UBE2D3 expression was a determinant factor in the progression of metastatic breast cancer. These two studies indicated that UBE2D3 expression is involved in cell cycle regulation via the degradation of cyclin D1; in consideration of this biological behavior, the present study proposes that UBE2D2 expression levels may promote tumor development. Furthermore, the current study identified that the expression of UBE2D3 was significantly lower in the esophageal cancer tissues compared with the adjacent healthy tissues, as well as significantly lower in the cancer tissues with lymph node involvement and poor differentiation. In addition, UBE2D3 appeared to be an independent prognostic factor for esophageal cancer. Thus, it is proposed that UBE2D3 expression may be involved in the progression of esophageal cancer.

Most notably, Spearman correlation coefficient analysis revealed a negative correlation between UBE2D3 and hTERT protein expression levels, which was consistent with a previous study (19). These results indicate that hTERT and UBE2D3 may interact with each other, validating the proposal that UBE2D3 potentially has a role in the hTERT signaling pathway. However, the mechanism by which the ubiquitination process of UBE2D3 is involved in the interaction with the hTERT pathway, and whether UBE2D3 expression exists as a universal phenomenon in all types of tumor, requires additional studies to be conducted in the future.

In conclusion, the present study demonstrated that hTERT and UBE2D3 expression are negatively correlated, and that the two proteins demonstrate a strong association with the prognosis in esophageal cancer. Furthermore, the expression of UBE2D3, lymph node involvement and tumor location were independent predictive prognostic factors; thus, UBE2D3 expression may be a promising prognostic biomarker in esophageal cancer. However, the current study was based on retrospective analysis and semi-quantitative research, therefore, prospective randomized clinical trials and basic quantitative research are required to evaluate the clinical utility of the present study results.

## Figures and Tables

**Table I:** Patient demographics and clinicopathological characteristics.

Characteristic	n (%)
Age, years	
<60	70 (46.7)
≥60	80 (53.3)
Gender	
Male	124 (82.7)
Female	26 (17.3)
Location	
Upper thoracic	21 (14.0)
Middle/lower thoracic	129 (86.0)
Tumor size, cm	
<5	81 (54.0)
≥5	69 (46.0)
Histological grade^a^	
G1/2	119 (79.3)
G3/G4	31 (20.7)
T stage	
T1–2	65 (43.3)
T3–4	85 (56.7)
N stage	
N0	76 (50.7)
N1–3	74 (49.3)
TNM stage	
Early (I–II)	88 (58.7)
Advanced (III–IV)	62 (41.3)
Adjuvant radiotherapy	
Yes	40 (26.7)
No	110 (73.3)
Adjuvant chemotherapy	
Yes	70 (46.7)
No	80 (53.3)
Recurrence	
No	66 (44.0)
Yes	84 (56.0)
Recurrence location	
Local-regional	50 (59.5)
Distant	34 (40.5)

a G1/2 were defined as well- or moderately-differentiated and G3/4 were defined as poorly-differentiated or undifferentiated.

TNM, tumor-node-metastasis.

**Table II:** Association between hTERT and UBE2D3 expression levels, and various clinicopathological characteristics.

	hTERT expression			UBE2D3 expression		
		
Characteristic	Low, n (%) (n=59)	High, n (%) (n=91)	P-value	Low, n (%) (n=95)	High, n (%) (n=55)	P-value
Age, years			0.067			0.651
<60	33 (47.1)	37 (52.9)		43 (61.4)	27 (38.6)	
≥60	26 (32.5)	54 (67.5)		52 (65.0)	28 (35.0)	
Gender			0.154			0.811
Male	52 (41.9)	72 (58.1)		78 (62.9)	46 (37.1)	
Female	7 (26.9)	19 (73.1)		17 (65.4)	9 (34.6)	
Location			0.900			0.261
Upper thoracic	8 (38.1)	13 (61.9)		11 (52.4)	10 (47.6)	
Middle/lower thoracic	51 (39.5)	78 (60.5)		84 (65.1)	45 (34.9)	
Tumor size, cm			0.039			0.262
<5	38 (46.9)	43 (53.1)		48 (59.3)	33 (40.7)	
≥5	21 (30.4)	48 (69.6)		47 (68.1)	22 (31.9)	
Histological grade^a^			0.032			0.007
G1/2	52 (43.7)	67 (56.3)		64 (57.1)	48 (42.9)	
G3/4	7 (22.6)	24 (77.4)		31 (81.6)	7 (18.4)	
T stage			0.030			0.154
T1–2	32 (49.2)	33 (50.8)		37 (56.9)	28 (43.1)	
T3–4	27 (45.8)	58 (63.7)		58 (68.2)	27 (31.8)	
N stage			0.018			0.016
N0	37 (48.7)	39 (51.3)		41 (53.9)	35 (46.1)	
N1–3	22 (29.7)	52 (70.3)		54 (73.0)	20 (27.0)	
TNM stage			0.012			0.347
Early (I–II)	42 (47.7)	46 (52.3)		53 (60.2)	35 (39.8)	
Advanced (III–IV)	17 (27.4)	45 (72.6)		42 (67.7)	20 (32.3)	
Recurrence			0.09			0.008
No	31 (47.0)	35 (53.0)		34 (51.5)	32 (48.5)	
Yes	28 (33.3)	56 (66.7)		61 (72.6)	23 (27.4)	
Recurrence location^b^			0.677			0.731
Local-regional	16 (32.0)	34 (68.0)		37 (74.0)	13 (26.0)	
Distant	12 (35.3)	22 (64.7)		24 (70.6)	10 (29.4)	

a G1/2 were defined as well- or moderately-differentiated and G3/4 were defined as poorly-differentiated or undifferentiated.

b Analysis was based on 84 recurrent cases of esophageal cancer.

hTERT, human telomerase reverse transcriptase; UBE2D3, ubiquitin-conjugating enzyme E2D 3; TNM, tumor-node-metastasis.

**Table III:** Univariate analysis of factors regarding overall survival.

Characteristic	n (%)	Five-year survival rate, %	χ^2^^a^	P-value
Age, years			0.459	0.498
<60	70 (46.7)	29.0		
≥60	80 (53.3)	22.0		
Gender			0.068	0.405
Male	124 (82.7)	28.0		
Female	26 (17.3)	17.0		
Location			4.455	0.035
Upper thoracic	21 (14.0)	11.0		
Middle/lower thoracic	129 (86.0)	30.0		
Tumor size, cm			3.812	0.051
<5	81 (54.0)	29.8		
≥5	69 (46.0)	20.2		
Histological grade^b^			0.068	0.795
G1/2	119 (79.3)	27.0		
G3/4	31 (20.7)	19.0		
T stage			4.278	0.039
T1–T2	65 (43.3)	35.5		
T3–T4	85 (56.7)	15.0		
N stage			11.290	0.001
N0	76 (50.7)	35.8		
N1–3	74 (49.3)	9.1		
TNM stage			16.000	<0.001
Early (I–II)	88 (58.7)	34.9		
Advanced (III–IV)	62 (41.3)	17.0		
Adjuvant radiotherapy			0.293	0.588
Yes	40 (26.7)	26.0		
No	110 (73.3)	23.0		
Adjuvant chemotherapy			0.004	0.951
Yes	70 (46.7)	26.1		
No	80 (53.3)	24.8		
hTERT expression			6.353	0.012
Low	59 (39.3)	35.0		
High	91 (60.7)	19.5		
UBE2D3 expression			9.145	0.002
Low	95 (63.3)	15.7		
High	55 (36.7)	40.9		

a Determined by performing a log-rank test.

b G1/2 were defined as well- or moderately-differentiated and G3/4 were defined as poorly-differentiated or undifferentiated.

TNM, tumor-node-metastasis; hTERT, human telomerase reverse transcriptase; UBE2D3, ubiquitin-conjugating enzyme E2D 3.

**Table IV:** Multivariate Cox proportional hazards analysis of overall survival.

	Overall survival		
	
Factor^a^	HR	95% CI	P-value
Tumor location	0.476	0.270–0.841	0.011
T stage	1.086	0.674–1.754	0.734
N stage	1.694	1.055–2.721	0.029
hTERT expression	1.589	0.990–2.551	0.055
UBE2D3 expression	0.487	0.293–0.807	0.005

a Factors were included according to the results of the univariate analysis.

HR, hazard ratio; CI, confidence interval; hTERT, human telomerase reverse transcriptase; UBE2D3, ubiquitin-conjugating enzyme E2D 3.
